# Early start of opicapone in Parkinson’s disease: evidence from a pooled analysis of phase 3 trials for sustained benefit in patients with recent onset of motor fluctuations

**DOI:** 10.3389/fneur.2025.1715748

**Published:** 2025-12-15

**Authors:** Joaquim J. Ferreira, Fabrizio Stocchi, Angelo Antonini, Olivier Rascol, Georg Ebersbach, Jaime Kulisevsky, Miguel M. Fonseca, Daniel Ramos, José Francisco Rocha, Helena C. Brigas, Joerg Holenz, Werner Poewe

**Affiliations:** 1 Laboratory of Clinical Pharmacology and Therapeutics, Faculdade de Medicina, Universidade Lisboa, Lisbon, Portugal; 2 CNS – Campus Neurológico, Torres Vedras, Portugal; 3 Institute for Research and Medical Care IRCCS San Raffaele, Roma, Italy; 4 San Raffaele University, Rome, Italy; 5 Neurodegenerative Disease Unit, Centre for Rare Neurological Diseases (ERN-RND), Department of Neuroscience, University of Padova, Padova, Italy; 6 IRCCS San Camillo, Venice, Italy; 7 NS-Park Network, Toulouse, France; 8 Department of Clinical Pharmacology and Neurosciences, Toulouse Parkinson Expert Centre, Toulouse NeuroToul Center of Excellence in Neurodegeneration (COEN), French NS-Park/F-CRIN Network, University of Toulouse 3, CHU of Toulouse, INSERM, Toulouse, France; 9 Movement Disorders Hospital, Beelitz-Heilstätten, Beelitz, Germany; 10 Movement Disorders Unit, Neurology Department, Sant Pau Hospital, Barcelona, Spain; 11 Biomedical Research Institute (IIB-Sant Pau), Barcelona, Catalunya, Spain; 12 Center for Networked Biomedical Research in Neurodegenerative Diseases (CIBERNED), Madrid, Spain; 13 BIAL - R&D Investments, S.A., Porto, Portugal; 14 BIAL – Portela & C^a^, S.A., Coronado, Portugal; 15 Department of Neurology, Medical University of Innsbruck, Innsbruck, Austria

**Keywords:** opicapone, motor fluctuations, Parkinson’s disease, clinical trial, long-term

## Abstract

**Background:**

Levodopa is the mainstay of Parkinson’s disease (PD) therapy, but its long-term use is often complicated by the development of motor fluctuations. While COMT inhibitors, such as opicapone, are routinely used for managing motor fluctuations, recent evidence has suggested that earlier vs. later intervention, at onset of motor fluctuations, may provide greater benefits. However, the longer-term impact of earlier intervention has not been well studied.

**Methods:**

This pooled subgroup analysis included data from randomized, 14–15 week, double-blind, placebo-controlled trials of opicapone and their 1-year open-label extensions. Exploratory analyses included all participants who were randomized to placebo or opicapone 50 mg, who continued opicapone into the open-label extension, and had developed motor fluctuations within 2 years before double-blind screening. Motor status was assessed using 24-h patient diaries and Unified Parkinson’s Disease Rating Scale (UPDRS) scores.

**Results:**

This post-hoc analysis included 227 patients who had been diagnosed with motor fluctuations within the prior 2 years (opicapone *n* = 117, placebo *n* = 110). Opicapone 50 mg significantly reduced daily OFF-time compared to placebo (mean placebo-adjusted reduction: –65.6 [95%CI, −105.5, −25.6] minutes, *p* = 0.0014) and increased Good ON-time (mean placebo-adjusted increase of: 88.3 [95% CI, 47.0, 129.6] minutes, *p* < 0.0001). Participants switching from double-blind placebo to open-label opicapone demonstrated the expected reductions in motor fluctuations. However, at the end of the open-label phase, those who started opicapone earlier maintained greater numeric reductions in OFF-time (mean treatment difference of −30.0 [95%CI, −74.8, 14.8] minutes, *p* = 0.2) and increases in total Good ON-time over 1 year (mean treatment difference of 38.2 [95%CI, −6.6, 82.9] minutes, *p* = 0.09) vs. those who switched from placebo. Mean levodopa doses remained stable throughout both the double-blind and open-label phases in both groups, indicating that the symptomatic benefits of opicapone were achieved without the need for increased levodopa dosing. There was no significant increase in troublesome dyskinesia.

**Conclusion:**

Early initiation of opicapone in PD patients with recently diagnosed motor fluctuations leads to sustained reductions in OFF-time and improvements in ON-time, without increasing dyskinesia or requiring escalation of levodopa doses. These exploratory findings support revising treatment strategies to include earlier use of opicapone, maximizing long-term benefits for patients with fluctuating PD.

## 1 Introduction

Levodopa remains the cornerstone of treatment for most people with Parkinson’s disease (PwP) (1) and is widely recommended as initial monotherapy for the majority of patients (2–4). Despite its unmatched efficacy, the long-term utility of the levodopa monotherapy approach is often restricted by the emergence of fluctuations. These fluctuations are characterized by alternating periods of symptom control and symptom recurrence (ON–OFF), and can significantly impact daily function and quality of life (5).

Wearing-off is typically one of the first types of fluctuation to appear (6). With continued treatment and as their disease progresses, many PwP find that standard levodopa regimens (three to four doses daily) no longer provide consistent symptom control throughout the day (7). This fluctuating pattern reflects levodopa’s short peripheral half-life and the progressive predominance of the ‘short-duration’ response (8–10). Current guidelines consider dopamine agonists, monoamine oxidase type B (MAO-B) inhibitors and catechol *O-*methyltransferase (COMT) inhibitors, as efficacious for adjunct use with levodopa to reduce motor fluctuations (2, 11). Among these, only COMT inhibitors enhance levodopa’s peripheral pharmacokinetics, providing more consistent bioavailability for crossing the blood–brain barrier and maintaining symptom control. Levodopa is routinely co-administered with a dopa decarboxylase (DDC) inhibitor (carbidopa or benserazide), which shifts its metabolism towards the COMT pathway (12). When combined with levodopa and a DDC inhibitor, COMT inhibitors increase levodopa’s half-life and bioavailability, while reducing the ‘pulsatile’ peak–trough fluctuations in plasma levels (13, 14).

Opicapone was rationally developed to meet the clinical need for an efficacious, well-tolerated, once-daily peripheral COMT inhibitor (15, 16). Recent evidence-based reviews note that it is the only COMT inhibitor with sufficient high quality evidence to be considered ‘effective’ in managing motor fluctuations (3). Such conclusions were primarily based on phase 3 randomized, double-blind, placebo-controlled trials of opicapone (17–19). Traditionally – partly because wearing-off was once held to be a complication of advanced disease (20) – COMT inhibitors have often been used in PwP with relatively advanced motor fluctuations (21). Accordingly, the opicapone pivotal studies (BIPARK-I and BIPARK-II) tended to enroll PwP who had well established motor fluctuations (17, 18). Recently however, observational trials and real world evidence has suggested that introducing opicapone at the onset of the first OFF symptoms may offer greater clinical benefit and improved tolerability to later disease (22–26), with superior efficacy to simply increasing the levodopa dose by 100 mg (27, 28).

We have previously reported results from pooled analyses of the pivotal double-blind studies which suggested that the placebo-adjusted reduction in OFF-time with opicapone 50 mg was greater in PwP who had developed motor fluctuations within the previous 2 years than in those with more established symptoms (29). Here we report on the long-term effectiveness of continued treatment for those PwP with early motor fluctuations (≤2 years) who continued into the 52-week open-label extension studies.

## 2 Materials and methods

BIPARK-I (17) and BIPARK-II (18) were randomized, double-blind, placebo-controlled trials of 14 to 15 weeks (visits 2 to 7), with full details previously published. Both studies were followed by open-label extensions that monitored patients for up to 1 year (end of open-label phase at visit 14) (18, 30). Institutional review boards at the participating sites provided ethics approval and the trials were done in accordance with the Declaration of Helsinki and International Conference on Harmonization Good Clinical Practice Guidelines. All patients provided written informed consent prior to conduct of any study related procedures.

### 2.1 Participants and study designs

Briefly, both trials enrolled adults aged 30–83 years with idiopathic Parkinson’s disease (PD) diagnosed for at least 3 years, and modified Hoehn and Yahr stage 1–3 during ON periods (17, 18). Eligible participants were required to be on 3–8 daily doses of levodopa for at least 1 year and to experience end-of-dose motor fluctuations with ≥1.5 h of OFF-time per day excluding morning akinesia to minimize variability and ensure accurate assessment of daytime OFF episodes.

Key exclusion criteria across both studies included a dyskinesia disability score >3 on the Unified Parkinson’s Disease Rating Scale (UPDRS) item 33, severe and/or unpredictable OFF periods, previous surgery or deep brain stimulation for PD. Patients with unstable cardiovascular or psychiatric conditions (e.g., major depression, dementia, impulse control disorders, suicidal ideation), significant liver disease, or transaminase levels >2 × ULN were excluded. Stable concomitant PD treatments were permitted, except for tolcapone, apomorphine (withdrawn ≥1 month prior), and entacapone (unless supplied for BIPARK-I).

Both double-blind studies assessed the safety and tolerability of opicapone 50 mg (taken in the evening, ≥1 h after the last dose of levodopa/DDCI) compared to placebo. Other doses studied (5 mg in BIPARK-I and 25 mg in both studies) were not relevant to this analysis (17, 18). BIPARK-1 also included an active comparator arm with entacapone, which is not relevant for this analysis [3].

Open-label phases commenced immediately after the double-blind phase and continued for 52 weeks. Patients began with opicapone 25 mg and could up-titrate to 50 mg for improved symptom control. In cases of dopaminergic adverse events (AEs), levodopa dosage was adjusted first, followed by opicapone down-titration if necessary (18, 30).

### 2.2 Outcomes

Patients were assessed at screening (visit 1), baseline (visit 2), week 1 (visit 3), between weeks 2 and 3 depending on need for levodopa adjustment (visit 4), followed by double-blind assessments after every 4 weeks until end of the double-blind phase (visit 7). Open-label phase assessments were at baseline (which could be the end of double-blind visit) and weeks 1, 4, 8, 16, 30, 46, and 52. Motor status was assessed throughout the double-blind and open-label phases using 24-h patient diaries (31) in which participants recorded their status: OFF, ON with troublesome dyskinesia, ON with non-troublesome dyskinesia, ON without dyskinesia, or asleep for every 30-min interval during the day for three consecutive days before each visit. Time spent in the OFF and ON states were calculated as the mean of the 3 preceding diary days to each visit, or the mean of available days if fewer than 3 days were recorded. Changes in ‘Good’ ON-time were calculated as the sum of ON-time without dyskinesia plus ON-time with non-troublesome dyskinesia (31).

Assessments of UPDRS Part II (activities of daily living [ADL] scores) and Part III (motor scores) (32), along with the Clinician’s Global Impression of Change (CGI-C) and the Patient’s Global Impression of Change (PGI-C), were conducted at each scheduled visit. Levodopa equivalent doses were estimated using the conversion formulae recommended by Jost et al. (33), and included opicapone (post-double-blind baseline). Safety outcomes for this subgroup of participants with early motor fluctuations will be reported separately.

### 2.3 Statistical analysis

This pooled *post-hoc* analysis was based on integration of individual participant data and included all participants (either study) who were randomized to either placebo or opicapone 50 mg who took ≥1 dose of study medication and had ≥1 post-baseline OFF-time assessment AND who continued with opicapone treatment into the open-label extension phases AND who had developed motor fluctuations (as judged by the investigator) within the 2 years prior to the double-blind screening visit.

Analyses were primarily descriptive (observed case). For the open-label phase, motor states were analyzed according to prior randomization category (i.e., opicapone [OPC]–OPC or placebo [PBO]–OPC). A Mixed Model for Repeated Measurements, modelling the change from baseline for each endpoint at each post-baseline visit, was used to estimate and compare the Least Square (LS) means by visit. The model included study, region, visit, and double-blind treatment as factors, with the double-blind baseline value as a continuous covariate. Interaction terms included treatment-by-visit, treatment-by-study, and treatment-by-region allowing the treatment effect to vary across visits, studies, and regions. Restricted maximum likelihood (REML) was employed to fit the model. The within-patient variation was modeled as a random effect with unstructured covariance matrix. The Kenward-Roger approximation was used to estimate the denominator degrees of freedom. Missing data was addressed through an MMRM under the Missing At Random (MAR) assumption, which incorporates all available observed data without imputation to provide information about the missing ones. This approach provides unbiased estimates when MAR holds by appropriately accounting for the correlated structure among repeated measures. Ninety-five percent confidence intervals and matching *p*-values were derived for LS means estimates by visits and the differences of OPC vs. PBO.

## 3 Results

### 3.1 Study population

**Figure 1:**
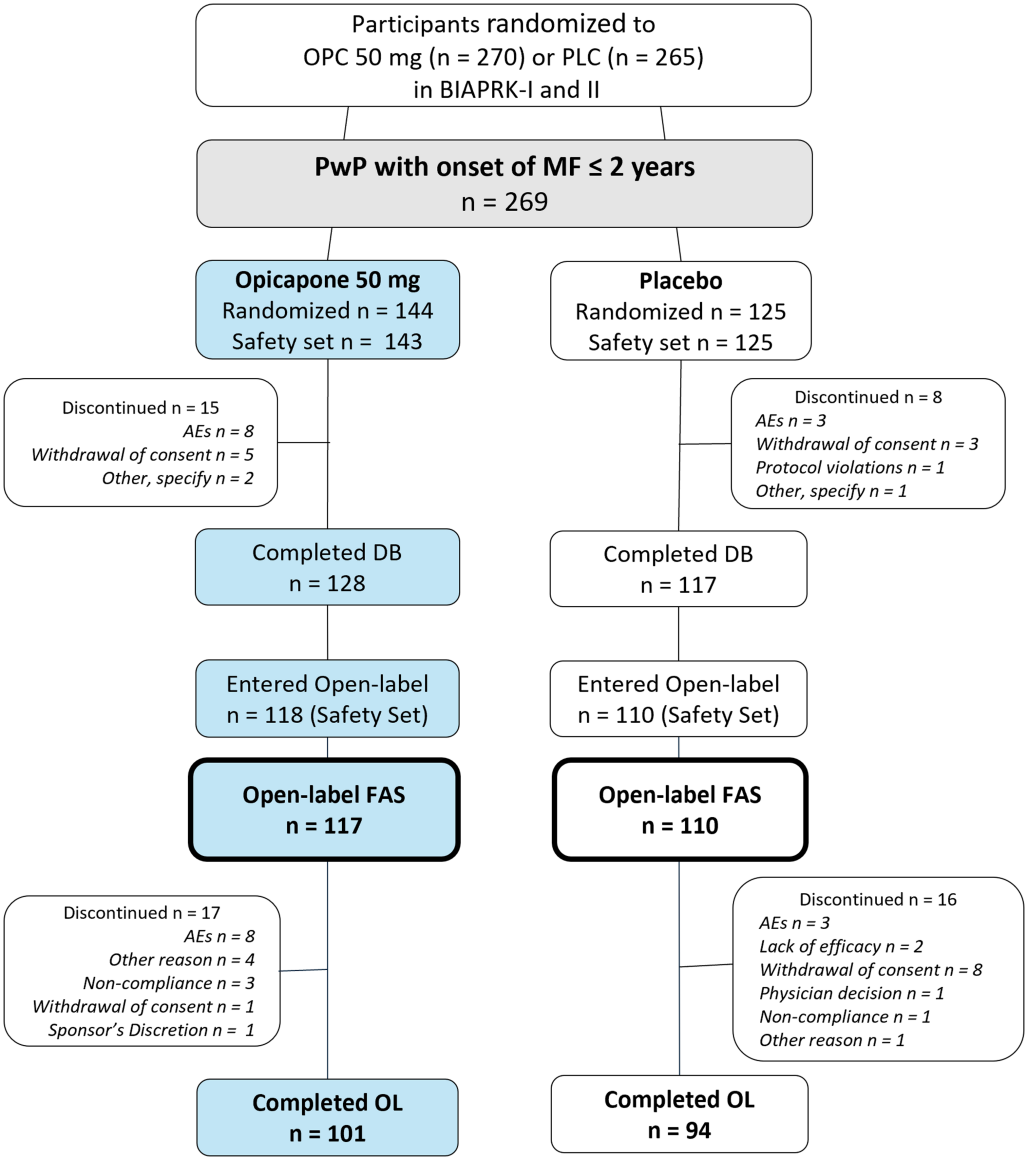
Patient disposition through the phase 3 double-blind and open-label trials.

Of the 535 participants randomized to receive placebo or opicapone 50 mg in BIPARK-I and -II, 269 had onset of motor fluctuations within the prior 2 years. Of these, 228 entered open label treatment and 227 were part of the Full analysis set (*n* = 117 for opicapone group and *n* = 110 for placebo group) (Figure 1); one patient previously treated with opicapone 50 mg in the double-blind phase did not take open-label treatment. Baseline demographic and clinical characteristics were generally balanced between the treatment groups at the start of the double-blind (Table 1). Despite a relatively recent diagnosis of motor fluctuations (1.0 years), participants experienced an average of 6.3 h of OFF-time when they entered the studies. Most participants were being treated with 3 to 5 levodopa doses at baseline, and most were treated with at least one concomitant PD medication.

**Table 1:** Participant demographics and clinical characteristics at double-blind baseline.

Parameter	Opicapone *n* = 117	Placebo *n* = 110
Demographics		
Male, *n* (%)	77 (65.8%)	62 (56.4%)
Age, years	64.6 ± 9.1	63.9 ± 8.8
Clinical history		
Time since PD diagnosis, years	5.9 ± 2.8	6.1 ± 2.7
Modified Hoehn & Yahr at ON, stage	2.4 ± 0.5	2.4 ± 0.5
UPDRS Part III (motor) score	25.6 ± 11.9	26.3 ± 12.0
UPDRS Part II (activities of daily living) score	17.0 ± 6.4	17.9 ± 6.8
Motor complication profile		
Time since motor fluctuation onset, years	1.0 ± 0.6	1.0 ± 0.6
Daily OFF-time, hours	6.4 ± 2.0	6.3 ± 2.1
Daily ON-time, hours	9.7 ± 2.1	10.0 ± 2.1
Daily Good ON-time, hours	9.5 ± 2.1	9.6 ± 2.0
ON-time without dyskinesia, hours	8.5 ± 2.8	8.0 ± 3.1
ON-time with troublesome dyskinesia, hours	0.2 ± 0.8	0.4 ± 1.0
Presence of dyskinesia, yes, *n* (%)	40 (34.2%)	40 (36.4%)
Levodopa use		
Levodopa duration, years	4.5 ± 3.0	4.9 ± 2.6
Levodopa dose at baseline, mg/day	638 ± 319	649 ± 305
Number of Levodopa intakes, *n* (%)		
3 intakes	35 (29.9%)	33 (30.0%)
4 intakes	37 (31.6%)	38 (34.5%)
5 intakes	26 (22.2%)	26 (23.6%)
6 intakes	9 (7.7%)	8 (7.3%)
≥ 7 intakes	10 (8.5%)	5 (4.5%)
LEDD^$^ at double-blind baseline, mg/day	755 ± 330	761 ± 332
LEDD^$^ at open-label baseline, mg/day	1.064 ± 470*^£^*	750 ± 312
Concomitant PD medication use		
Levodopa monotherapy*, *n* (%)	23 (19.7%)	22 (20.0%)
Dopamine agonists, *n* (%) Pramipexole Ropinirole Piribedil Rotigotine	81 (69.2%) 45 (38.5%) 26 (22.2%) 7 (6.0%) 3 (2.6%)	73 (66.4%) 37 (33.6%) 30 (27.3%) 4 (3.6%) 2 (1.8%)
MAO-B inhibitor, *n* (%) Rasagiline Selegiline	23 (19.7%) 16 (13.7%) 7 (6.0%)	19 (17.3%) 11 (10.0%) 8 (7.3%)
Amantadine, *n* (%)	27 (23.1%)	20 (18.2%)
Anticholinergics, *n* (%)	5 (4.3%)	11 (10.0%)

Data are mean ± SD or *n* (%). Good ON-time was calculated as the sum of ON-time without dyskinesia plus ON-time with non-troublesome dyskinesia. ^$^LEDD calculated considering MAO-B inhibitors and dopamine agonists. ^£^Considering OPC for LEDD calculation. *Without dopamine agonists, MAO-B inhibitors, amantadine or anticholinergics.

### 3.2 Double-blind phase

**Figure 2:**
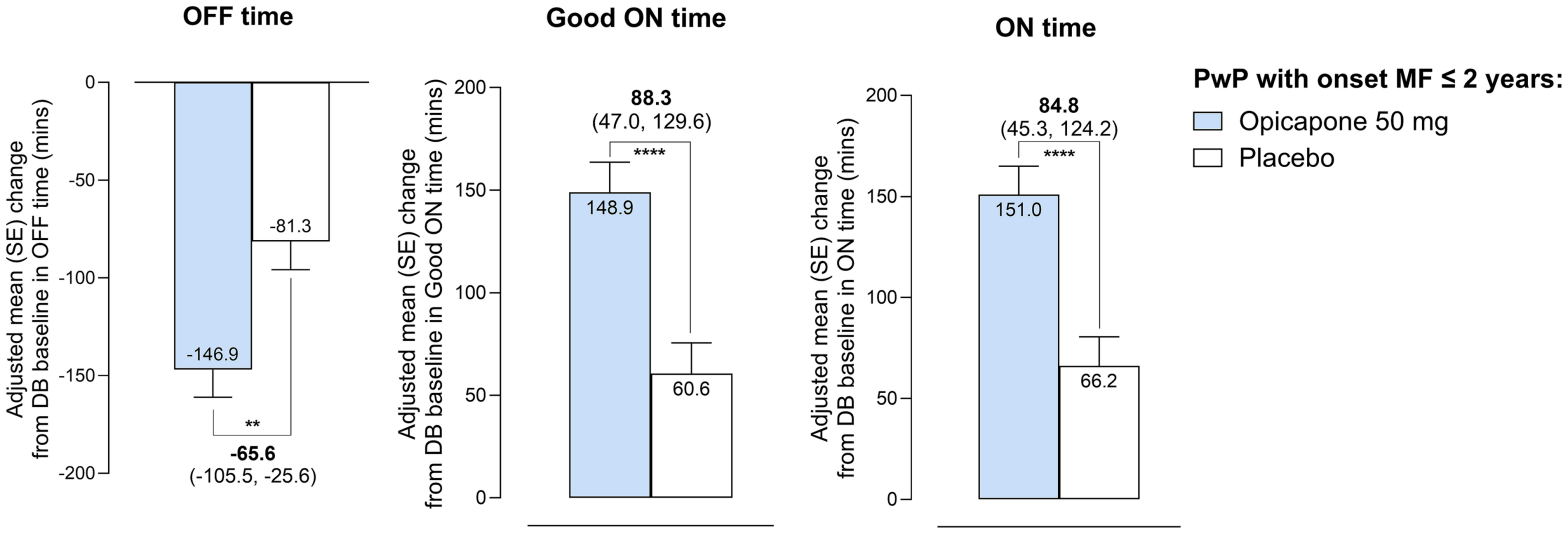
Changes in motor status, as assessed by 24-h patient diaries, during the double-blind phase in absolute OFF-time, Good ON-time and ON-time in PwP with onset of motor fluctuations ≤ 2 years. **p ≤ 0.01; ****p ≤ 0.0001.

Treatment with opicapone 50 mg significantly reduced absolute daily OFF-time in participants with a recent diagnosis of motor fluctuations. The LS mean [95% CI] placebo-adjusted treatment effect was −65.6 [−105.5, −25.6] minutes (*p* = 0.0014), in favor of opicapone (Figure 2; Supplementary Table 1). Reductions in OFF-time were mirrored by significant increases in Good ON-time (placebo-adjusted treatment effect of 88.3 [47.0, 129.6]) and in ON-time (placebo-adjusted treatment effect of 84.8 [45.3, 124.2]).

Significant differences favoring opicapone treatment were observed for UPDRS ADL scores (LS mean placebo-adjusted treatment difference of −1.4 [−2.6, −0.2], *p* = 0.02). Although non-significantly different, UPDRS motor scores also tended to favor opicapone treatment (−1.3 [−3.1, 0.5], *p* = 0.2) (Supplementary Table 1). PGI-C and CGI-C results also favored opicapone treatment with more participants reporting an improvement with active treatment (67.5% vs. 55.0% with placebo for PGI-C and 70.1% vs. 51.8% with placebo for CGI-C) (Supplementary Table 1).

### 3.3 Open-label phase

**Figure 3:**
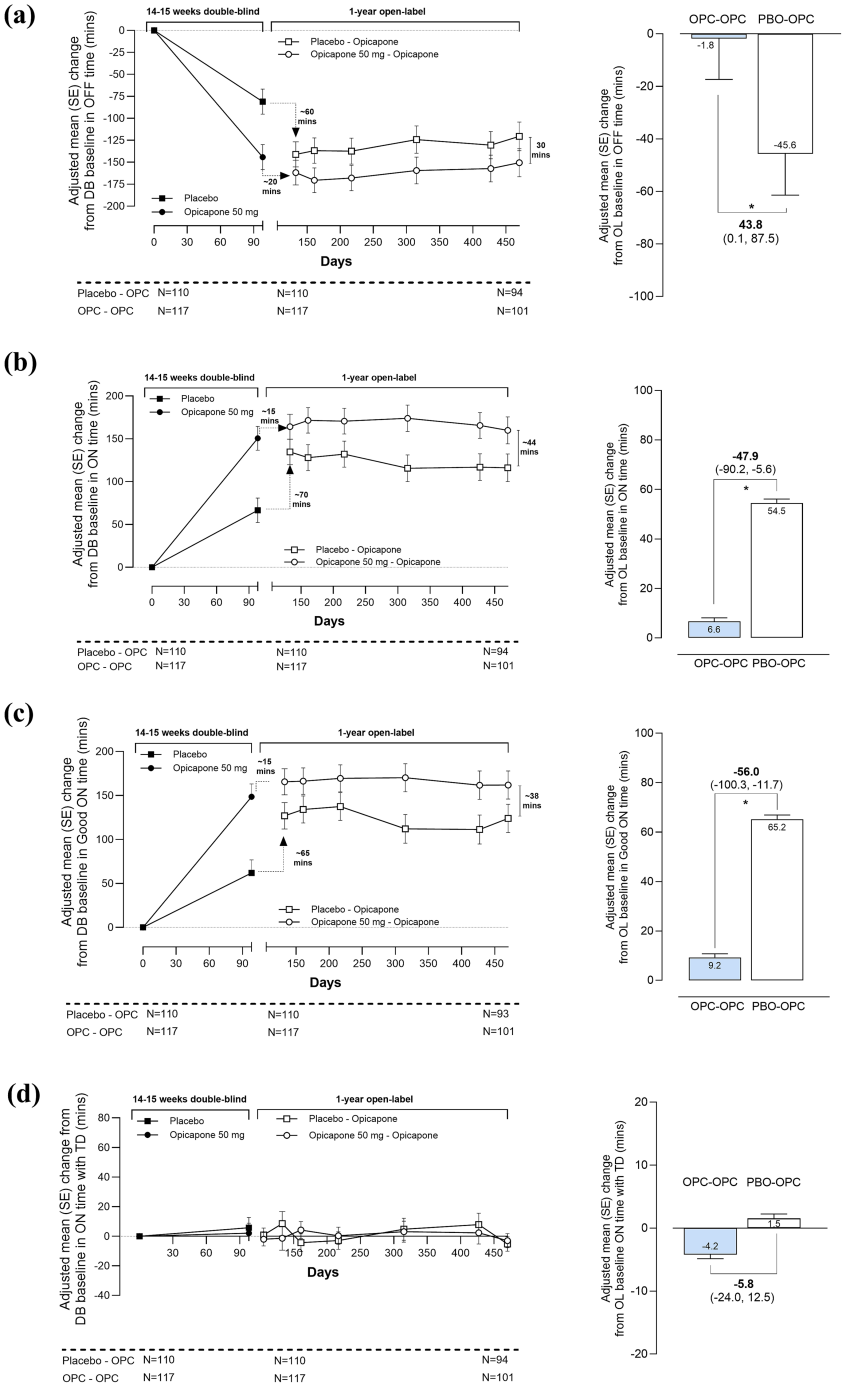
Long-term effectiveness of opicapone across open-label treatment, by prior randomization **(a)** OFF-time **(b)** ON-time **(c)** Good ON-time **(d)** ON-time with troublesome dyskinesia in PwP with onset of motor fluctuations ≤ 2 years. *p ≤ 0.05. OPC, opicapone; PBO, placebo.

Participants switching from double-blind placebo to open-label opicapone demonstrated the expected mean reduction in OFF-time (−58.8 [−98.1; −19.4] minutes) and increase in ON-time (67.7 [28.3, 107.0] minutes) after around 4 weeks of open-label treatment (Figure 3). After 1-year open label opicapone treatment, participants in the PBO-OPC group had an adjusted mean ± SE reduction of -45.6 ± 15.8 min of OFF-time (−120.5 ± 16.3 min vs. double-blind baseline) and a mean increase of 54.5 ± 15.3 min of ON-time (116.2 ± 16.1 min vs. double-blind baseline). For participants in the OPC-OPC group, patient diary results indicated a maintenance of symptomatic effect. At the end of the open-label phase, participants in the OPC-OPC group showed a mean additional reduction of −1.8 ± 15.5 min spent in the OFF state accompanied by an additional 6.6 ± 15.0 min of ON-time versus open label baseline (−150.5 ± 15.9 min of OFF-time and 159.9 ± 15.8 min of ON-time vs. double-blind baseline).

Of note, and despite the significant benefits seen upon switching treatment, participants in the PBO-OPC group never achieved the full magnitude of benefits observed in the OPC-OPC group. At the end of open label follow-up, participants in the OPC-OPC group tended to have less time in the OFF state than those in the PBO-OPC group (mean treatment difference of −30.0 [−74.8, 14.8] minutes versus double-blind baseline, *p* = 0.2). Likewise, participants in the OPC-OPC group spent significantly more time in the ON state than those in the PBO-OPC group (mean treatment difference of 43.8 [−0.7, 88.2] minutes versus double-blind baseline, *p* = 0.05). Results for Good ON-time were similar to total ON-time, indicating that most of the increase in ON-time was of good quality (Table 2). Taken overall, no relevant changes were observed in the mean ON-time with troublesome dyskinesia (Bad ON-time) during the open-label phase. However, participants who had already developed dyskinesia at double-blind baseline showed a more variable pattern (large standard error sizes) across the open-label treatment period than those who did not suffer dyskinesia at baseline (Supplementary Figure 1). The long-term maintenance of clinical effect was confirmed by CGI-C and PGI-C data. At the end of the open-label period a majority of participants rated as improved relative to double-blind baseline (Supplementary Table 1).

**Table 2:** Long-term effectiveness of opicapone by prior randomization.

Parameter	Change from DB baseline		Change from OL baseline	
	OPC-OPC *N* = 117	PBO-OPC *N* = 110	OPC-OPC *N* = 117	PBO-OPC *N* = 110
OFF-time (min)				
Adjusted mean change from baseline	−150.5 ± 15.9	−120.5 ± 16.3	−1.8 ± 15.5	−45.6 ± 15.8
Difference at Visit 14 (95% CI)	−30.0 (−74.8, 14.8)		43.8 (0.1, 87.5)	
*p*-value	0.1885		**0.0494**	
ON-time (min)				
Adjusted mean change from baseline	159.9 ± 15.8	116.2 ± 16.1	6.6 ± 15.0	54.5 ± 15.3
Difference at Visit 14 (95% CI)	43.8 (−0.7, 88.2)		−47.9 (−90.2, −5.6)	
*p*-value	0.0534		**0.0267**	
Good ON-time (min)				
Adjusted mean change from baseline	162.0 ± 15.9	123.9 ± 16.2	9.2 ± 15.7	65.2 ± 16.1
Difference at Visit 14 (95% CI)	38.2 (−6.6, 82.9)		−56.0 (−100.3, −11.7)	
*p*-value	0.0943		**0.0135**	
ON-time with Troublesome dyskinesia (min)				
Adjusted mean change from baseline	−3.1 ± 4.8	−5.3 ± 4.9	−4.2 ± 6.5	1.5 ± 6.7
Difference at Visit 14 (95% CI)	2.2 (−11.3, 15.8)		−5.8 (−24.0, 12.5)	
*p*-value	0.7465		0.5349	

Data are LS mean ± SE. *p* values based on MMRM analyses. Good ON-time was calculated as the sum of ON-time without dyskinesia plus ON-time with non-troublesome dyskinesia. Bold values indicate statistical significance.

**Figure 4:**
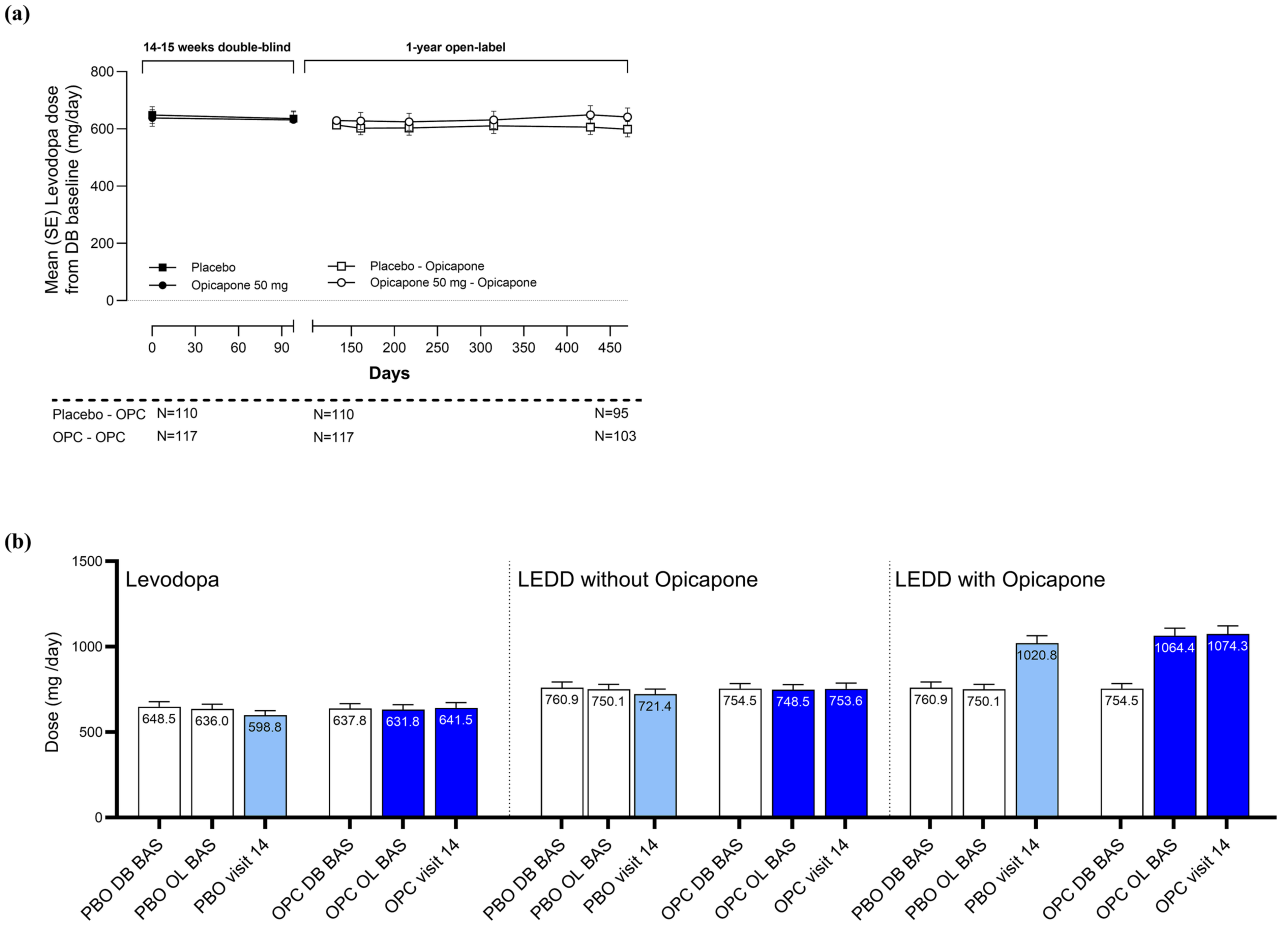
Daily levodopa dosing over time **(a)** levodopa dosing across double-blind and open-label phases **(b)** levodopa and levodopa equivalent daily doses at double-blind and open-label baselines and end of open-label phase.

At the group level, mean levodopa doses remained stable in both the OPC-OPC and PBO-OPC throughout the double-blind and open label phases (Figure 4). In the OPC-OPC group, mean ±SD levodopa doses were: 638 ± 319 mg at double-blind baseline, 632 ± 310 mg at open-label baseline, and 642 ± 317 mg at the end of open-label study (visit 14). In the PBO-OPC group mean ±SD levodopa doses were: 649 ± 305 mg at double-blind baseline, 636 ± 282 mg at open-label baseline, and 599 ± 260 mg at the end of open-label study (visit 14). However, more participants who switched from placebo to opicapone changed their levodopa dose compared with those who began treatment with opicapone (27.3% in the PBO-OPC group changed levodopa dose vs. 18.8% in the OPC-OPC group). The main reason for changing levodopa daily dose was disease progression (17.1% in the OPC-OPC group and 26.4% in the PBO-OPC group) followed by adverse events (1.7% in the OPC-OPC group and 0.9% in the PBO-OPC group). The percentage of dopamine agonists and MAO-B inhibitors was overall similar over time. Levodopa equivalent daily dose (LEDD) changes during the study reflected the addition of opicapone (Figure 4).

## 4 Discussion

This *post-hoc* subgroup analysis of pivotal trial data for opicapone confirms its long-term symptomatic efficacy as an adjunct to levodopa in PwP who have developed motor fluctuations within the past 2 years, effectively challenging the traditional positioning of COMT inhibition as a treatment for established fluctuations. Notably, participants who switched from placebo to opicapone failed to achieve the full magnitude of benefit observed in those who initiated opicapone earlier, suggesting that earlier treatment may offer greater advantages for patients with recent onset of motor fluctuations. By the end of the open-label treatment phase, participants who had been treated with opicapone throughout had an average reduction in OFF-time of 151 min (>2.5 h) accompanied by an average increase in ON-time of 160 min (>2.5 h). Results were similar for Good ON-time and there were no relevant changes in the time spent with troublesome dyskinesia. Of note, participants were largely maintained on their current levodopa doses over the entire study period and clinical global impression data also confirmed the long-term maintenance of clinical effect.

Participants with a shorter documented history of motor fluctuations appeared to derive greater benefit from opicapone 50 mg than reported for the overall cohort (OFF-time reductions of 66 min at end of double-blind treatment vs. 58 min overall cohort (34)). This is also consistent with the findings of Rocha et al., who reported consistently greater short-term efficacy in ‘earlier’ versus ‘later’ disease when categorized according to disease duration, disease stage, and baseline PD medications (29). Additionally, this early subgroup showed small but significant improvements in activities of daily living and numeric improvement in motor symptoms that are not always expected in a group already treated with levodopa and with motor complications due to lower sensitivity in this population. The merits of earlier versus later initiation of COMT inhibition in early wearing-off has long been discussed based on *post-hoc* analyses, where early rather than delayed use of the COMT inhibitor entacapone provided a modest clinical benefit that was maintained for up to 5 years (35). Our data indicate that patients who started opicapone earlier after diagnosis of motor fluctuations sustained their initial improvements over time with small incremental gains over 1 year, while those who began treatment later (by 14–15 weeks) still benefited meaningfully from opicapone initiation (~60 min), but without fully reaching the magnitude of effect observed in the early-start group at study end. This is a difficult concept to study under controlled conditions, however there is growing real-world evidence supporting the use of opicapone in patients experiencing early motor fluctuations. For example, López-Ariztegui et al. reported data from over 1,000 patients treated in Spain, indicating that opicapone is particularly beneficial for individuals in the earlier stages of Parkinson’s disease with mild motor fluctuations (25). López-Manzares et al. recently published findings from a Spanish registry cohort of early fluctuators (defined as motor fluctuation onset within ≤2 years), showing a comparable magnitude of reduction in OFF-time with long-term opicapone treatment – from 3.8 h at baseline to 1.9 h at 12 months (22). Furthermore, a pooled analysis of two observational studies by Ferreira et al. demonstrated that adjunctive opicapone 50 mg was significantly more effective in reducing OFF-time than the commonly used strategy of adding an extra 100 mg dose of levodopa (28). These findings have led national expert panels to recommend opicapone for patients with PD who present with early motor fluctuations (23, 36).

Another commonly used proxy for effectiveness is levodopa dosing, where stable maintenance suggests a sustained symptomatic benefit. Despite open-label investigators having the flexibility to adjust treatment based on clinical need, levodopa doses remained stable – further supporting the long-term efficacy of opicapone in these patients (26, 37). It is also notable that while the need for dosing changes remained low, more participants in the PBO-OPC group had to change their levodopa dosing during open-label treatment. This might, in part, reflect the fact that patients in the OPC-OPC group had already been on their regimen for 14–15 weeks. However, the main reason for dosing change in both groups over 1.3 years was ‘disease progression’. In a recent resource utilization study conducted in the UK, PwP treated with opicapone as their first-line COMT inhibitor reduced their LEDD by approximately 26% during the first year of treatment and then stabilized. This was in contrast to PwP treated with entacapone who showed an initial decrease in the first 6 months but a gradual increase thereafter (38). Current therapeutic approaches to PD treatment are centered around keeping the levodopa dose as low as possible for as long as possible to avoid the development of troublesome dyskinesia (39). Here, it is pertinent to note that addition of opicapone did not significantly increase time spent ON with dyskinesia or troublesome dyskinesia, as most of OFF-time was translated into a Good ON-time.

The primary strength of this pooled analysis lies in the consistency of study designs and the use of individual patient-level data. However, longer follow-up is needed to evaluate the persistence of the levodopa ‘dose stabilizing’ effect in PwP with early fluctuations. Our observations are constrained by the retrospective, post-hoc nature of the analyses, and the open-label data remain vulnerable to selection, attrition, and confounding biases due to lack of placebo control. In addition, the onset of motor fluctuations was judged by the investigators based on medical records, and several studies show that clinicians often underestimate the presence of motor fluctuations (e.g., due to mild severity of OFF in the early stages or lack of awareness of the full range of OFF symptoms) and the onset of wearing-off occurs earlier than previously thought. Indeed, underscoring the difficulty of early identification, patients in this study already had around 6 h of daily OFF-time. It has also been reported that PwP often have problems distinguishing when they transition to a dyskinetic state (40). However, while this might affect the accuracy of overall dyskinesia emergence using patient diaries, it is unlikely to affect the reporting of troublesome dyskinesia which is inherently defined by the PwP. While patient diaries and clinical scales remain standard, such issues highlight the potential of wearable technologies for objective, continuous, quantitative monitoring (41).

## 5 Conclusion

In conclusion, this *post-hoc* subgroup analysis reinforces the long-term efficacy of opicapone in reducing OFF-time and increasing ON-time, without a corresponding rise in dyskinesia among PwP experiencing recently emerging motor fluctuations. Adding adjunct opicapone allowed PwP with recent onset of motor fluctuations to maintain their existing levodopa and adjunct medication (including dopamine agonists and MAOB inhibitor) regimens. These findings broaden the evidence base for COMT inhibition across all stages of fluctuating PD, suggesting a potential benefit for adding opicapone earlier (within 2 years of motor fluctuations onset). Prospective, long-term studies in PwP with early fluctuations are warranted. Our observations support the importance of recognizing wearing-off symptoms early and optimizing treatment promptly to maximize long-term benefits.

## Data Availability

The original contributions presented in the study are included in the article/Supplementary material, further inquiries can be directed to the corresponding author.
