# Supplementary material for: Early start of opicapone in Parkinson’s disease: evidence from a pooled analysis of phase 3 trials for sustained benefit in patients with recent onset of motor fluctuations
Source: Front Neurol. 2025 Dec 15;16:1715748. doi: 10.3389/fneur.2025.1715748 (PMC12745269; doi:10.3389/fneur.2025.1715748)

Supplementary Material

Table e1. Efficacy outcomes for participants with recent onset of motor fluctuations (≤2 years) at end of double-blind studies

	**Opicapone 50 mg N=117**	**Placebo N=110**
**OFF-time** (min)		
Adjusted mean ± SE change from baseline	-146.9 ± 14.2	-81.3 ± 14.5
Treatment difference vs. Placebo (95% CI)	-65.6 ± 20.3 (-105.5, -25.6)	
p-value for opicapone 50 mg vs. Placebo	**0.0014**	
**ON-time** (min)		
Adjusted mean ± SE change from baseline	151.0 ± 14.0	66.2 ± 14.3
Mean difference ± SE vs. Placebo (95%CI)	84.8 ± 20.0 (45.3, 124.2)	
p-value for opicapone 50 mg vs. Placebo	**<0.0001**	
**Good ON-time** (min)		
Adjusted mean ± SE change from baseline	148.9 ± 14.7	60.6 ± 15.0
Mean difference ± SE vs. Placebo (95%CI)	88.3 ± 21.0 (47.0, 129.6)	
p-value for opicapone 50 mg vs. Placebo	**<0.0001**	
**UPDRS Part II (ADL) scores**		
Adjusted mean ± SE change from baseline	-3.8 ± 0.4	-2.4 ± 0.4
Mean difference ± SE vs. Placebo (95%CI)	-1.4 ± 0.6 (-2.6, -0.2)	
p-value for opicapone 50 mg vs. Placebo	**0.025**	
**UPDRS Part III (motor) scores**		
Adjusted mean ± SE change from baseline	-4.7 ± 0.7	-3.4 ± 0.7
Mean difference ± SE vs. Placebo (95%CI)	-1.3 ± 0.9 (-3.1, 0.5)	
p-value for opicapone 50 mg vs. Placebo	0.1645	
**PGI-C**		
Participants with improvement[a], n (%)	79/117 (67.5%)<br>73/113 (64.6%)[b]	60/109 (55.0%)<br>73/105(69.5%)[b]
**CGI-C**		
Participants with improvement[a], n (%)	82/117 (70.1%)<br>74/113 (65.5%)[b]	57/110 (51.8%)<br>72/105 (68.6%)[b]

Data are LS mean ± SE; P values based on MMRM analyses; Good ON-time was calculated as the sum of ON-time without dyskinesia plus ON-time with non-troublesome dyskinesia [a]reporting minimal, much or very much improved. [b]end of open-label

Figure e1. Change in ON time with troublesome dyskinesia according to the presence or absence of dyskinesia at double-blind baseline (a) no dyskinesia reported (b) presence of dyskinesia reported

a)

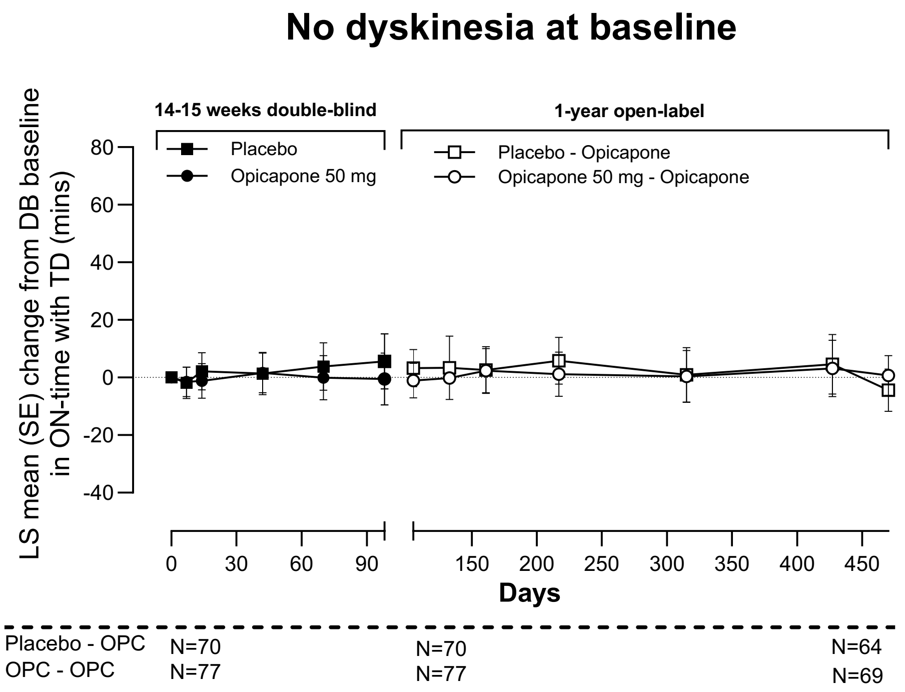

b)

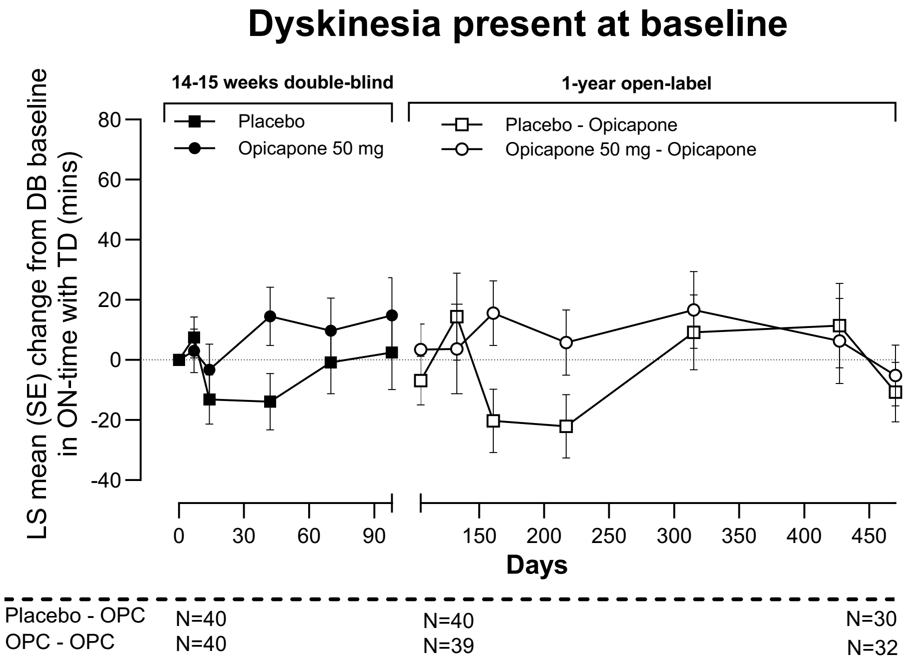

Supplement: Supplementary file 1 [file Data_Sheet_1.pdf]
